# Editorial: Insights in neuro-oncology and neurosurgical oncology: 2021

**DOI:** 10.3389/fonc.2022.1121113

**Published:** 2023-01-17

**Authors:** Erik P. Sulman, David D. Eisenstat

**Affiliations:** ^1^ Section of Neuro-oncology & Neurosurgical Oncology, Frontiers in Oncology and Frontiers in Neurology, Lausanne, Switzerland; ^2^ Department of Radiation Oncology, NYU Grossman School of Medicine, New York, NY, United States; ^3^ Brain and Spine Tumor Center, Laura and Isaac Perlmutter Cancer Center, New York NY, United States; ^4^ Department of Radiation Oncology, NYU Langone Health, New York, NY, United States; ^5^ Children’s Cancer Centre, Royal Children’s Hospital, Parkville, VIC, Australia; ^6^ Department of Stem Cell Biology, Murdoch Children’s Research Institute, Parkville, VIC, Australia; ^7^ Department of Paediatrics, University of Melbourne, Parkville, VIC, Australia

**Keywords:** brain tumor, glioblastoma, brain metastases, immune therapy, gene therapy, CAR T cells, tumor heterogeneity

The Frontiers Research Topic titled ***Insights in Neuro-Oncology and Neurosurgical Oncology: 2021*** includes a collection of 18 articles published from May 2021 to March 2022. The topics summarize our understanding of as well as key advances in the field of Neuro-Oncology, covering a variety of subjects focused on primary central nervous system (CNS) tumors, such as biomarkers and diagnostics, model systems, anatomic and surgical considerations, and novel approaches to therapeutics. A comprehensive review of brain metastases is also included.

## Insights into biomarkers and diagnostics in neuro-oncology

In 2018, Capper et al. introduced DNA methylation profiling for CNS tumors, significantly improving our capacity to correctly categorize and diagnose brain tumors in children and adults (1). More recently, the fifth edition of the World Health Organization (WHO) Classification of Tumours of the Central Nervous System was published, integrating tumor histology, tumor grade (where applicable), tumor markers, and molecular genetics (2).

Zreik et al. studied disparities in the reporting of 1p/19 co-deletions (codel) in oligodendroglial tumors before and after the introduction of the fourth version of the WHO CNS classification in 2016 through an analysis of the National Cancer Database. Interestingly, the reported rate of codel testing increased from ~45% in 2011 to nearly 60% in 2017. Furthermore, those with a reported test result received adjuvant therapy with an OR of 1.73. However, significant disparities were observed by geography as well as ethnicity and race.

Cell surface markers are characterized in two separate studies. Lu et al. investigate the impact of tumor purity and focus on the role of CD3E, a novel immune biomarker, in the tumor microenvironment of low-grade gliomas (LGG) of adults using the TCGA and GEO databases, with validation in a real-world cohort of 100 Asian patients. The prognostic utility of the cell surface expressed and well-described glioma stem cell marker, CD133, in adult high-grade gliomas (HGG) is assessed in a systematic review provided by Shadbad et al. The authors conclude that using a 10% cut-off, overexpression of CD133 protein was associated with a very poor progression-free survival (PFS) linked to tumor progression/recurrence.

In an interesting report, Prasad et al. describe a multi-generational family with germline BAP1-inactivating mutations resulting in meningiomas with rhabdoid features. Rhabdoid meningiomas are classified as WHO Grade III meningiomas and have a variable prognosis. Germline mutations of the tumor suppressor gene BAP1 are linked to a rare tumor predisposition syndrome and affected patients are at very high risk of melanoma and mesothelioma. The authors recommend that germline testing be offered for patients with meningiomas harboring BAP1-inactivating mutations.

## Neuroanatomic and neurosurgical insights

In this group of six manuscripts, insights were provided into diverse topics regarding specific neuroanatomic considerations, imaging adjuncts, and localized therapies. Skardelly et al. **s** assess the extent of resection (EOR) and residual tumor volume in a retrospective, multicenter cohort study of adult patients with glioblastoma (GBM). The authors developed a nomogram validated in a separate patient cohort that can be applied in clinical practice and incorporated into prospective non-randomized clinical trials where EOR could introduce bias in the outcomes concerning PFS and overall survival (OS). Feng et al. apply Enhanced Recovery After Surgery (ERAS) principles regarding rehabilitation, quality of life, and survivorship in 50 brain tumor patients experiencing craniotomy. Although postoperative recovery was enhanced in this patient cohort, the authors recommend multicenter collaborative studies in order to confirm that ERAS can enhance patient prognosis while concomitantly reducing postoperative complications.

Surgical approaches to tumors located in in or originating from the fourth ventricle were the focus of Onorini et al., who studied 92 consecutive pediatric patients treated at a single center by one pediatric neurosurgeon who used either telovelar (51 patients) or transvermian (41 patients) surgical approaches to tumor resection. In this single-center study, a relatively low rate (11%) of cerebellar mutism (also referred to as posterior fossa syndrome) was noted, and there were no significant differences between either surgical strategy. The authors advocate training in and the application of either neurosurgical approach to tumors localized to the fourth ventricle, individualized to tumor anatomy, infiltration of the vermis, and lateral or upwards extension. Li et al. discuss advances in the neurosurgical approach to parasellar meningiomas, incorporating preoperative imaging and protection of the cerebral arteries and their perforating branches, which can be compressed, encased, or, rarely, invaded by these extra-axial tumors. The authors promote the use of a bidirectional dissection technique.

Gao et al. present an interesting manuscript regarding glioma-associated epilepsy (GAE) and the emerging application of radiomics in neuro-oncology in a cohort of 166 adult patients with frontal gliomas. In addition to identifying 17 specific MR imaging features, the authors also consider the influence of patient age and tumor grade in an integrated clinical-radiomics predictive model.

The application of laser interstitial thermal therapy (LITT) as a minimally invasive adjunct to surgery is explored by Noh et al.. During LITT, continuous real-time temperature mapping was conducted using magnetic resonance thermometry. The authors studied 17 patients, paying specific attention to the contribution of signal dropout, an artifact of biopsy that often precedes LITT. Within this group, 6 of the 17 patients had biopsies with artifacts due to the presence of blood or air that affected the thresholds of thermal damage at tumor borders.

## Considerations on pathogenesis and disease modeling

Two publications included in this article collection focus on GBM tumor heterogeneity and the subventricular zone (SVZ), respectively. Comba et al. explore spatiotemporal heterogeneity in glioblastomas in a timely review of the influence of the molecular genetic features of the tumor, the tumor microenvironment (including non-transformed neuronal and glial cells, immune, mesenchymal, and stem cells), and dynamic qualities within the tumor itself. The authors discuss the contributions of more recently adopted platforms including machine learning in histopathology, single-cell transcriptomics, and spatial transcriptomics. Data acquisition using these technologies will better inform our preclinical models with translational implications, including high-throughput drug screening.

Beiriger et al. compare neural stem/precursor cells (NSC) resident to the adult SVZ and glioma stem cells (GSC), which are both implicated in the pathogenesis of gliomas. The authors review several *in vitro* and *in vivo* model systems and include human brain and/or GBM organoids as important emerging model systems in the study of the contributions of NSC and GSC, thereby reframing our approaches toward improving preclinical translational research in the laboratory.

## Considerations for the use of tumor treating fields in neuro-oncology

Two articles discuss insights into the efficacy of tumor treating fields (TTF), an FDA-approved treatment adjunct for adults with glioblastoma, which is being explored for other treatment indications in children and adults with brain and other solid tumors. The effect of the corticosteroid dexamethasone (DXM) on TTF is addressed by Linder et al. The authors use GBM cell lines treated with DXM and either radiotherapy or TTF *in vitro.* In addition, they perform a retrospective analysis of GBM patients who received TTF +/- DXM. The authors conclude that concomitant DXM with TTF did not affect the efficacy of TTF *in vitro* or in clinical practice. The impact of the ongoing COVID-19 pandemic on the use of TTF is the focus of a separate contribution by Gatson et al., who summarize an expert panel discussion, which took place during the early months of the pandemic. Since TTF is administered by a portable device that is used in the home and is not known for additional immunosuppressive effects over and above the intrinsic effects of the tumor itself, the authors recommend that specific patient populations, especially the elderly and those with co-morbidities, may benefit from TTF-mediated therapies.

## Treatment advances using viral gene, immune checkpoint, and CAR T-cell mediated therapies

A major focus in neuro-oncology reflects ongoing efforts to advance our understanding and utilization of therapies that harness the immune system. Viral-mediated gene therapies for GBM are reviewed by Li et al. The authors summarize several viral vectors and their potential or current use in viral gene therapy, including retroviruses, lentiviruses, adenoviruses, herpes simplex virus (HSV), and oncolytic viruses; many of these vectors are under active investigation in clinical trials in neuro-oncology. Combined immune checkpoint inhibitor therapy applied to meningeal melanoma, a relatively uncommon tumor entity belonging to the class of primary melanocytic tumors of the CNS, is presented by Burgos et al.

Burns et al. summarize current efforts in the laboratory and the clinic to use CAR T-cell therapies, which have already been adopted for some hematopoietic malignancies expressing CD19, in the treatment of very challenging pediatric brain tumors, especially diffuse midline gliomas with Histone H3K27 alterations, which are relatively prevalent in children. Methods of application (intraventricular, intra-tumoral, intravenous), CAR T-cell design, target identification, and characterization, targeting one or more antigens (i.e., multivalent CARs), cytokine release syndrome and other CAR T-cell associated toxicities, and combinatorial therapies with kinase and other small molecule inhibitors and/or immune checkpoint inhibitors are all under active investigation.

## Insights into brain metastases

Finally, Li et al. provide a comprehensive review of brain metastases (intracranial metastatic disease or IMD) in adults for which the application of improved molecular genetics-based diagnostics, neuroimaging, minimally invasive surgery, novel local therapies, improvements in radiation therapy (stereotactic radiosurgery, hippocampal sparing whole brain radiotherapy, etc.), neurocognitive rehabilitation strategies, and the application of either targeted and/or immunotherapies offer some hope to those patients with very poor prognoses overall. Specific sections of this review article focus on breast cancer, NSCLC, and melanoma, given their relative contributions to IMD in adults.

## Concluding remarks

Despite significant progress in neuro-oncology translational and clinical research, we have yet to fully realize the potential of improved diagnostic platforms; advances in therapy from surgery; radiation; and other local or systemic therapies, including targeted, viral gene, or immunotherapies. Given the high priority of this combined effort, the neuro-oncology community has mapped out an ambitious agenda for improving both the duration and quality of survival for our pediatric and adult patients with primary or secondary tumors of the central nervous system. However, we will have to continue our collective journey to achieve these worthy goals.

## Author contributions

ES and DE conceptualized and co-wrote the manuscript. Both authors approved the final version.
